# Role of Tau Protein Hyperphosphorylation in Diabetic Retinal Neurodegeneration

**DOI:** 10.1155/joph/3278794

**Published:** 2025-03-12

**Authors:** Jingyu Mu, Zengrui Zhang, Chao Jiang, Haoming Geng, Junguo Duan

**Affiliations:** ^1^Eye School of Chengdu University of TCM, Chengdu, Sichuan, China; ^2^Key Laboratory of Sichuan Province Ophthalmopathy Prevention & Cure and Visual Function Protection with TCM Laboratory, Chengdu, Sichuan, China; ^3^Retinal Image Technology and Chronic Vascular Disease Prevention & Control and Collaborative Innovation Center, Chengdu, Sichuan, China; ^4^College of Life and Health Sciences, Institute of Neuroscience, Northeastern University, Shenyang, China; ^5^Ineye Hospital of Chengdu University of TCM, Chengdu, Sichuan, China

**Keywords:** abeta, diabetic retinal neurodegeneration, diabetic retinopathy, retinal ganglion cells, tau

## Abstract

Diabetic retinal neurodegeneration (DRN) is an early manifestation of diabetic retinopathy (DR) characterized by neurodegeneration that precedes microvascular abnormalities in the retina. DRN is characterized by apoptosis of retinal ganglion cells (involves alterations in retinal ganglion cells [RGCs], photoreceptors, amacrine cells and bipolar cells and so on), reactive gliosis, and reduced retinal neuronal function. Tau, a microtubule-associated protein, is a key mediator of neurotoxicity in neurodegenerative diseases, with functions in phosphorylation-dependent microtubule assembly and stabilization, axonal transport, and neurite outgrowth. The hyperphosphorylated tau (p-tau) loses its ability to bind to microtubules and aggregates to form paired helical filaments (PHFs), which further form neurofibrillary tangles (NFTs), leading to abnormal cell scaffolding and cell death. Studies have shown that p-tau can cause degeneration of RGCs in DR, making tau pathology a new pathophysiological model for DR. Here, we review the mechanisms by which p-tau contribute to DRN, including insulin resistance or lack of insulin, mitochondrial damage such as mitophagy impairment, mitochondrial axonal transport defects, mitochondrial bioenergetics dysfunction, and impaired mitochondrial dynamics, Abeta toxicity, and inflammation. Therefore, this article proposes that tau protein hyperphosphorylation plays a crucial role in the pathogenesis of DRN and may serve as a novel therapeutic target for combating DRN.

## 1. Background

Diabetic retinopathy (DR) is a severe blinding eye disease that is a complication of diabetes. The global prevalence of DR among diabetes patients was 22.27%, while in China it was 18.45%, leading to a significant economic burden [1, 2]. Increasing evidence suggested that diabetic retinal neurodegeneration (DRN) was an additional component of DR. Some studies considered DRN as an early stage of DR, and the retinal neurodegeneration in DR may occur before vascular abnormalities [3]. DRN usually has no obvious clinical symptoms, but optical coherence tomography (OCT) can reveal thinning of the retina, including the retinal nerve fiber layer (RNFL), ganglion cell layer (GCL), and inner plexiform layer (IPL) [4–6]; visual functional impairments, such as contrast sensitivity, visual field examination, and dark-adapted visual sensitivity [7–9]; as well as retinal electroretinogram changes [10]. DRN's pathological manifestations include early apoptosis of neurons, decreased synaptic proteins in retinal nerve terminals [11], and abnormalities in glial cells [12]. The molecular biology mechanisms of DRN may involve imbalances in neurotrophic and cytokine factors, reactive glial cells, changes in signal pathways and cell adhesion molecules, activation of cysteine proteases, and mitochondrial dysfunction [13].

In both animal experiments and clinical studies, the early detection of retinal ganglion cells' (RGCs) death or loss in DR has been observed, leading to functional impairment and degeneration of retinal neuronal cells [14–17]. Researchers have also found that in early-stage diabetes, high blood sugar can lead to morphological changes in RGCs' dendritic terminals, such as increased length, density, and quantity [18]. At 24–26 weeks after the onset of diabetes, a reduction in photoreceptors in the outer nuclear layer of the retina can be observed [19, 20]. Furthermore, studies have found the loss of dopaminergic and cholinergic amacrine cells in the early stages of DR, and the loss of these neurons might play a critical role in the development of diabetic visual impairment [21]. Apoptosis and loss of RGCs, morphological alterations in surviving RGCs, reduction of photoreceptors, and loss of amacrine cells are possible causes of retinal functional abnormalities (photoreceptors, bipolar, amacrine, and ganglion cell components) in diabetes. Another early characteristic of diabetes-induced retinal neurodegeneration is the activation and dysfunction of neuroglial cells, with Müller glial cells being the primary neuroglial cells in the retina [22, 23]. Small glial cells in the retina are also activated, producing inflammatory factors that exacerbate neuroglial dysfunction [24]. The impact of high blood sugar on retinal neurons may trigger early visual defects in diabetes [25, 26].

Some studies discovered mitochondrial swelling in RGCs and reduced superoxide dismutase (SOD) activity in the retina of rats induced with high blood sugar after 4 weeks using streptozotocin (STZ), indicating that oxidative stress may lead to RGCs loss [27]. Another study indicated that STZ-induced diabetes exacerbated RGCs functional impairment and loss in the glaucoma DBA/2 J mouse model, accompanied by increased glial reactivity and elevated expression of oxidative stress markers, IL-6 and TNF-α, showing that diabetes can promote oxidative stress and inflammation-mediated RGCs' functional impairment and glial reactivity [28]. Brain-derived neurotrophic factor (BDNF) is present in Müller glial cells and RGCs in the retina. In animal models of diabetes, BDNF can exert neurotrophic effects by preventing RGC and amacrine cell death [29, 30]. In diabetes, high blood sugar can lead to retinal neurodegeneration through various biochemical pathways, including inflammation, oxidative stress, or imbalance of retinal neuroprotective factors [14, 31].

The microtubule-associated protein tau (MAPT), when bound to microtubule protein, serves as the core for early microtubule assembly, promoting the extension and aggregation of other microtubule proteins to form microtubules. This prevents disassembly and maintains the stability of the microtubule structure. It is also a crucial mediator of neurotoxicity in neurodegenerative diseases, with functions in phosphorylation-dependent microtubule assembly and stabilization, axonal transport, and neurite outgrowth. The hyperphosphorylated tau (p-tau) disrupts its binding to microtubules, leading to the aggregation of paired helical filaments (PHFs) and the subsequent formation of neurofibrillary tangles (NFTs). This process damages the stability of the cellular microtubule cytoskeleton, interferes with axonal transport, and causes mitochondrial dysfunction, synaptic abnormalities, and loss, ultimately leading to neurodegeneration [32, 33]. In recent years, some scholars have proposed that the hyperphosphorylation of tau protein may be related to the occurrence and development of DRN [34], and there was relatively little research on treatment methods for DRN. This article will discuss the potential mechanisms involved in the hyperphosphorylation of tau protein in DRN and new strategies for early detection and treatment.

## 2. Tau Gene

**Figure 1:**
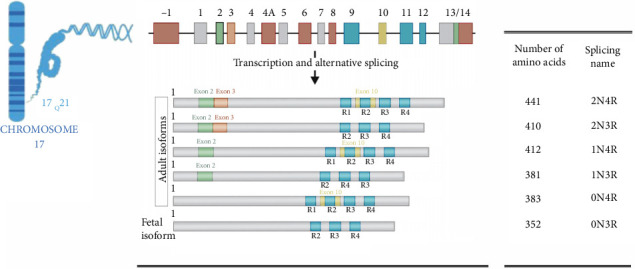
Organization of the tau gene, and the resulting six isoforms of the protein.

The human tau protein is encoded by a single gene called MAPT, located on chromosome 17 (17q21.1) (Figure 1). It produces three transcripts (2, 6, and 8 kb), with the 2 kb transcript encoding nuclear tau [35], and the 6 and 9 kb transcripts transcribe axonal tau. The 6 kb transcript encodes tau proteins expressed in both the central nervous system (CNS) and the peripheral nervous system (PNS). On the other hand, the 9 kb transcript encodes tau proteins expressed in the retina and the PNS. In addition, the 2 kb transcript is widely expressed throughout various tissues [36–38]. The MAPT gene consists of 16 exons, of which 11 encode the tau protein [39]. Exon 1 is a promoter that is transcribed but not translated, and although Exon 14 is present in mRNA, it does not code for protein. Exons 1, 4, 5, 7, 9, 11, 12, and 13 are constitutive splicing exons expressed in the adult CNS and PNS [38, 40–43]. Exons 2, 3, and 10 can undergo alternative splicing [44], with Exon 3 being expressed only in conjunction with Exon 2 [41, 42], and Exon 10 being spliced in the CNS but expressed in the PNS [38]. Exon 4A is expressed only in the PNS and retina in the adult (refer to Table 1) [36]. In the human CNS, six isoforms of tau protein (0 N3R, 1 N3R, 2 N3R, 0 N4R, 1 N4R, and 2 N4R) can be generated due to selective splicing of Exons 2, 3, and 10 [45–47], where “R” represents the number of microtubule repeats and “N” represents the number of N-terminal inserts. The differences among the isoforms depend on the number of repeats in their C-terminal tandem repeat domains. The splicing pattern of Exon 2 and the inclusion of Exon 3 determine whether the isoform has zero (0N), one (1N), or two (2N) N-terminal inserts, each consisting of 29 amino acids [47]. The splicing pattern of Exon 10 determines whether the tau protein contains four (4R-tau) or three (3R-tau) microtubule repeats, each consisting of 31 or 32 amino acids [41, 42, 47, 48]. The six isoforms are differentially expressed in various types of neurons and at different stages of brain development due to selective splicing [44, 49].

## 3. Structural Features of Tau Protein

The normal structure of tau protein is crucial for its proper functioning. The primary structure of tau protein is divided into four functional regions from the carboxy-terminal to the amino-terminal end: the projection domain at the N-terminus, the proline-rich domain, the microtubule-binding domain (MBD), and the C-terminal domain [47, 50]. The N-terminal domain is rich in negatively charged residues, which allow the tau protein to separate different microtubules through electrostatic repulsion [51]. The proline-rich domain and the MBD, which contain multiple amino acid receptor residues, participate more in the interaction with different signal proteins. These signal proteins can either be folded by tau protein or change the conformational state and activity of tau protein itself [52].

The C-terminal domain not only plays a critical role in regulating microtubule assembly induction and interacting with the plasma membrane but also generates overall molecular asymmetry, with its negative charge contributing to microtubule spacing function [53–57]. Studies have shown that the C-terminal domain exerts its spacing function through long-range repulsive forces that are ionic strength-dependent [53]. Although the repeat domains are mainly responsible for microtubule growth, the C-terminal region significantly affects microtubule growth and nucleation processes [55]. In addition, the C-terminal domain's interaction with the plasma membrane is phosphorylation-dependent [56], and while C-terminal phosphorylation alone does not significantly affect microtubule assembly, when combined with proline-rich region modifications, it can alter tau's conformational properties and membrane cortex interactions [56, 57]. Tau protein is a naturally unfolded protein with no stable secondary or tertiary structure, exhibiting an irregularly coiled structure [58]. However, when interacting with other proteins or ligands (such as microtubules), tau protein can undergo local folding [59]. The quaternary structure of tau protein can form dimers/trimers, oligomers, and larger polymers [60]. Dimers/trimers and oligomers may exhibit toxicity [61], with cysteine residues participating in the formation of tau dimers and oligomers [62, 63]. Tau oligomers (TauOs) are likely the most neurotoxic form of tau. In tau-related disorders, TauOs play a crucial pathological role and are considered abnormal forms that contribute significantly to neuronal loss and behavioral impairments. The mechanisms underlying the neurotoxicity of TauOs can be further explored in detail through the review written by Niewiadomska G and colleagues [64]. *In vitro*, tau can be induced to form protofibrils by oxidizing its two cysteine residues, Cys-322 and Cys-291, generating intermolecular disulfide bonds that promote dimerization and protofibril formation. Cys-322 inhibits the formation of tau aggregates *in vivo*, and blocking Cys-322 mutation can significantly eliminate tau toxicity. Blocking Cys-291 mutation does not seem to affect the conformation or aggregation tendency of tau, but the toxicity of the mutant protein is lower than that of the wild-type (WT) protein [65]. However, research has indicated that [66] the elimination of Cys322 by mutation significantly inhibits the phosphorylation of tau protein at disease-associated sites. In contrast, regulating its redox state did not impact the phosphorylation status. Preventing cysteine oxidation has no effect on tau protein phosphorylation, at least at the investigated sites, suggesting that these two types of protein modifications are independent of each other [67]. Related studies have shown that oligomeric tau caused neurotoxicity, which may mainly come from TauOs (o.tau) [68–70], which were considered as temporary intermediates in the process of tau aggregation [71]. RNA and TIA1 (RNA binding protein) promote tau oligomerization [72]. TauOs (o.tau) play a key role in neuronal loss and behavioral disorders in tauopathies, the most common of which is Alzheimer's disease (AD). In addition, glycosylation of tau protein leads to the formation of larger aggregates similar to NFTs [73].

## 4. The Position and Physiological Functions of Tau

Tau protein is a member of the microtubule-associated proteins' (MAPs) family, which can act as the core of microtubule assembly in the early stages by binding to microtubule proteins, promoting the extension and aggregation of other microtubule proteins on this core, preventing disassembly, and maintaining the stability of their structure. It also plays an important role in neuronal plasticity [74]. Under physiological conditions, tau protein is mainly distributed in neurons in the human brain [75], with a small amount found in oligodendrocytes, astrocytes, and extracellular fluid [76]. In addition to the CNS, tau protein is also expressed by peripheral neurons [77]. Within neurons, tau protein is mainly distributed in the axon [52], while only a small amount is found in the dendritic region (including the cell membrane, nucleus, and mitochondria) [78, 79]. Tau protein can affect axonal transport and growth, neuronal polarization, and therefore influence neurons and the brain [58, 80–82]. Tau protein may regulate axonal transport by affecting the function of motor proteins, dynein, and kinesin, which transport cargoes to the microtubule's negative end (toward the cell body) and positive end (toward the axon terminal) [83]. Among the neuronal MAPs, tau is predominantly localized to the axonal compartment. Therefore, tau protein may play specific roles in axon growth and maintenance of the axonal structure and function [84], while tau is mainly located in axons during neuronal maturation and polarization [82]. Under pathological conditions, tau protein abnormally assembles into insoluble aggregates in both neurons and glial cells, leading to synaptic dysfunction and neuronal cell death. This series of neurodegenerative diseases caused by the aggregation of tau protein is collectively known as tauopathies or tau diseases [47, 85].

In recent years, a unique form of tau protein has been identified—*cis*-p-tau, a *cis*-isomer of p-tau. This form has been shown to drive neurotoxicity and neurodegeneration, serving as an early driver in several neurodegenerative diseases, including AD, traumatic brain injury (TBI), chronic traumatic encephalopathy (CTE), as well as vascular contributions to cognitive impairment and dementia (VCID) [86]. Research has shown that in mouse models of acute TBI and *in vitro* neuronal stress, neurons generate large amounts of *cis*-p-tau, which disrupts the axonal microtubule network and mitochondrial transport, and spreads to other neurons, triggering apoptosis. This unique conformation of tau protein is referred to as “cistauosis,” and it appears before the onset of other types of tauopathies. *Cis*-tauopathy is an early precursor to the tauopathies previously described and serves as an early marker of neurodegeneration, which can be blocked by *cis*-antibody [87].

## 5. Posttranslational Modifications (PTMs) of Tau

PTM of tau protein is an important way to regulate the structure and function of tau protein. These modifications are usually catalyzed by enzymes and involve adding chemical groups, sugars, or proteins to specific residues of the target protein. Common modifications include phosphorylation, acetylation, glycosylation, truncation, ubiquitination, methylation, isomerization of aspartic acid residues, and others [88]. Tau protein hyperphosphorylation can lead to RGCs' dysfunction and synaptic loss in diabetes retina, and then result in DRN [34]. For understanding tau PTM, please refer to Wang, Ye, and other reviews [58, 89].

Phosphorylation is the most common form of posttranslational modification of tau protein, and it mainly occurs on 85 amino acid residues (45 serine residues, 35 threonine residues, and 5 tyrosine residues), which are potential phosphorylation sites [90]. Tau phosphorylation is a physiological process and regulates the microtubule assembly of tau protein in many interactions to maintain the dynamic properties of microtubules under normal physiological conditions. However, the tau protein found in PHFs was hyperphosphorylated [91]. Hyperphosphorylation of tau protein results in the loss of its ability to bind to microtubules, leading to aggregation and formation of PHFs, which ultimately lead to abnormal cell skeleton and cell death, and finally, neurodegeneration in various neurological diseases (e.g., DRN) [32, 34]. Recent studies have shown that NFTs themselves may not be toxic, while hyperphosphorylated soluble TauOs may be the culprit in neurotoxicity [92, 93]. Indications proposed that TauOs negatively impact synapses by decreasing the levels of synaptophysin and oxidoreductase, septin-11, which were synaptic vesicle–associated proteins, and by disturbing mitochondria through reducing the levels of NADH–ubiquinone electron transport chain complex I simultaneously, TauOs can impair fast axonal transport and influence learning and memory [94]. In many tau protein kinases, the imbalance between tau protein kinases and phosphatases can mediate the excessive phosphorylation of tau, and an increase in protein kinase activity or a decrease in phosphatase activity is a direct cause of excessive phosphorylation of tau protein [33]. Tau protein kinases include glycogen synthase kinase-3β (GSK3β), cyclin-dependent kinase 5 (CDK-5), mitogen-activated protein kinase/extracellular signal–regulated kinase (MAPK/ERK), c-Jun N-terminal kinase (JNK), protein phosphatase 2A (PP2A), cyclic AMP–dependent protein kinase (PKA), and Ca2+- or calmodulin-dependent protein kinase II (CaMKII), among others [47, 90, 95, 96]. In addition, there was evidence that the PI3K/AKT/GSK3 signaling pathway is one of the main pathways that regulate the excessive phosphorylation of tau protein [97].

Tau glycosylation mainly includes two types: N-glycosylation and O-GlcNAc glycosylation. N-glycosylation is the connection between the peptide chain of the protein and the glycosidic bond between the terminal N-acetylglucosamine (GlcNAc) and the NH2 group of asparagine. O-GlcNAc glycosylation is the process of O-acetyl-glucosamine attaching to serine, threonine, or tyrosine residues [98, 99]. N-glycosylation is prone to occur on hyperacetylated tau, while unmodified tau can undergo O-GlcNAc glycosylation [100]. O-GlcNAc glycosylation requires the participation of two enzymes, O-GlcNAc glycosyltransferase (OGT) and O-GlcNAc hydrolase (OGA). The process involves OGT adding O-GlcNAc to proteins, while OGA is responsible for the dynamic process of removing O-GlcNAc. Studies found that the phosphorylation of tau was reduced in the cortex and hippocampus of rats receiving OGA inhibitors [101]. In addition, O-GlcNAc glycosylation of tau was significantly lower in brain tissues of AD patients compared to the control group, and it was negatively correlated with phosphorylated tau [102]. Therefore, O-GlcNAc glycosylation may prevent tau from excessive phosphorylation or reduce neurofibrillary tangle formation [103]. Regulating the level of tau protein glycosylation to reduce excessive phosphorylation may become a potential therapeutic approach for neurodegenerative diseases. Therefore, the correlation between PTM of tau protein and DRN needs further research.

## 6. Mechanisms of Retinal Neurodegeneration Induced by Diabetes

Hyperglycemia and impaired insulin signaling are considered major factors in the retinal pathology of DR [104]. Retinal nerve damage due to diabetes mellitus is controlled by a variety of mechanisms, including glutamate excitotoxicity, neurotrophic factor imbalance, oxidative stress, inflammation, and glial reactivity [105, 106]. These glial reactivities are closely related to the proliferation, activation, and regression of Müller cells, microglia, and astrocytes in diabetic retinas, resulting in the production of inflammatory factors and the loss of retinal protective factors. This leads to reactive gliosis, causing functional impairment and apoptosis of retinal neurons. Such glial reactivities may play a decisive role in the onset and progression of DRN [107–109]. In animal models of T2DM induced by a high-fat/high-sugar diet, impaired insulin-like growth factor-1 (IGF-1) signaling, increased GSK-3β activity, and p-tau have been observed in the brain [110, 111]. GSK-3β, a key tau kinase, contributes to neurodegeneration by directly promoting tau hyperphosphorylation through its activation [112]. Furthermore, insulin resistance leads to a significant decrease in the phosphorylation of Akt and GSK3β, and a significant increase in the phosphorylation of tau, which is a marker of neurodegenerative diseases, in brain/neuron-specific insulin receptor knockout (NIRKO) mice [113].

Recent findings in the pathophysiology of DRN indicate that galectin-3, serine racemase (SRR), and p-tau play critical roles in driving DRN [114]. Studies have shown that knockout of galectin-3 or inhibition of SRR can alleviate DRN [115, 116]. Tau is a key mediator of neurotoxicity in neurodegenerative diseases (e.g., AD) and has not been previously studied in relation to DRN. In a high-fat diet (HFD)–induced diabetic mouse model study, p-tau was found not only to cause visual defects and synaptic loss in RGCs but also to accelerate retinal neurodegeneration with glucolipotoxicity-induced synapse loss in RGCs by impairing mitochondrial transport and reducing mitochondrial activity in a GSK3β-dependent manner. This aberrant tau protein aggregation further triggers retinal microangiopathy and induces RGCs apoptosis [34]. Based on the above mechanisms, it was found that topical ocular administration of GLP-1 receptor agonist liraglutide can effectively block p-tau–induced retinal neurodegeneration through activation of the GLP-1 R/Akt/GSK3β signaling pathway, showing promise as a potential strategy for the treatment of early diabetic retinal tauopathies [117]. Similarly, ginsenoside Rg1 (GRg1) was found to be able to prevent tau-mediated RGCs synaptic neurodegeneration through activation of the IRS-1/Akt/GSK3β signaling pathway, providing important clues for exploring novel therapeutic targets for DRN [118]. The above studies collectively reveal the key role of signaling pathways that regulate tau phosphorylation in the treatment of DRN.

Although there is currently no direct evidence of tau aggregation in the human retina after diabetes mellitus, it is noteworthy that a recent study detected p-tau in the RGC layer, which was associated with retinal neurological dysfunction in human tau (hTau) transgenic mice carrying the P301S mutation, revealing that p-tau is closely associated with RGC degeneration [119]. In addition, researchers are increasingly recognizing that diabetes and AD share many common pathological features. One study found that, compared to patients with macular holes, the vitreous fluid of DR patients had significantly higher levels of tau protein [120]. Diabetic animal models exhibit brain changes similar to those seen in AD, including increased tau phosphorylation and synaptic degeneration [95, 121]. Some studies even suggest that p-tau was a significant pathological marker of dementia in patients with diabetes [122, 123]. Studies also indicate that in a HFD–induced diabetic RGCs neurodegeneration model, p-tau was mediated by GSK3β activation, which leads to visual function defects in RGCs by impairing synaptic and mitochondrial function. This further supports the notion that retinal tauopathy can serve as a novel pathophysiological model for DRN [34]. In addition, disruption of the insulin signaling pathway or defects in glucose metabolism can induce p-tau [111, 113, 124]. Mitochondrial dysfunction, oxidative stress, inflammation, and overactivation of tau-related kinases are also key mechanisms involved in p-tau induction in diabetes [125–127]. Although these studies reveal an association between diabetes and tau phosphorylation, the specific mechanisms by which p-tau occurs in the pathogenesis and progression of DRN still require further investigation and clarification.

## 7. The Pathogenic Role of p-Tau in DRN

The synaptic degeneration and axonal injury of RGCs that occur before RGCs apoptosis may be the earliest event in the development of DR [34]. The retina is part of the CNS, including photoreceptors, horizontal cells, amacrine cells, bipolar cells, and RGCs. Under pathological conditions, tau is hyperphosphorylated and self-aggregates, leading to the pathogenic conformation of neurodegenerative diseases, known as tauopathy such as AD and Parkinson's disease [50]. Recently, some scholars have proposed that retinal tauopathy was a new pathophysiological model for DRN. The p-tau destroys the synapses and mitochondrial function of RGCs, leading to DRN; furthermore, they found that in the retina of HFD–induced diabetic mice, p-tau at S396/S404 and T205/T231 sites could trigger synaptic degeneration [34]. The current research indicated that p-tau has been identified as a crucial toxic mediator in synaptic neurodegeneration of RGCs in diabetes [117]; the following will summarize how p-tau may lead to neuronal degeneration through mechanisms such as mitochondrial damage, Aβ toxicity, and inflammation, which are involved in the occurrence and development of DRN.

### 7.1. Mitochondrial Damage

Related studies have shown that mitochondrial damage may be involved in the occurrence and development of DRN [128–130]. Mitochondria plays a key role in neuronal survival and death by regulating energy metabolism and apoptotic pathways, and their damage and dysfunction are associated with neurodegenerative diseases [131–133]. Simultaneously, some scholars have suggested that under diabetes-induced metabolic stress, abnormal GSK-3β activation drives p-tau and downregulation of β-catenin, resulting in synaptic neurodegeneration preceding mitochondrial impairment and apoptosis of RGCs [134]. In addition, the p-tau can also cause mitochondrial damage, such as damaged mitochondrial autophagy, mitochondrial axonal transport defects [135], mitochondrial bioenergetic disorders, and impaired mitochondrial dynamics [136], leading to neuronal degeneration, so it can participate in the occurrence and development of DRN. Thus, p-tau may be involved in the occurrence and development of DRN through mitochondrial damage.

#### 7.1.1. Mitophagy

During the process of mitophagy, damaged mitochondria are recognized by the isolation membrane (phagophore), which then isolates them into a double-membraned structure of mitochondria [137, 138]. These structures then fuse with lysosomes, leading to the degradation of the mitochondria. Mitophagy can remove damaged or dead mitochondria through autophagic clearance, reducing the accumulation of damaged mitochondria and exerting a positive protective effect on neurons. Studies have shown that pathological Aβ and p-tau can impair mitophagy, leading to an increase in damaged mitochondria and the initiation of a malignant cycle of self-replication [139]. The PTEN-induced PINK1/Parkin pathway is a common pathway for mitophagy. PTEN-induced putative kinase (PINK1) protein kinase phosphorylates Parkin (an E3 ubiquitin ligase) protein, causing it to selectively recruit to the outer membrane of damaged mitochondria with high expression. It is then degraded in the lysosome, thus mediating mitophagy [140].

According to studies, Parkin can protect and promote mitochondrial autophagy in RGCs to combat excitotoxicity [141, 142]. Devi TS found that under high glucose induction, Parkin aggregates in the mitochondria of early DR retinal Müller cell line (rMC1), and then induces mitochondrial autophagy [143]. At the same time, researchers found that notoginsenoside R1 (NGR1) can alleviate Müller cell damage in the retina by enhancing PINK1–Parkin–dependent mitochondrial autophagy [144]. In neurons overexpressing NH2htau, abnormal binding of Parkin and ubiquitin carboxy-terminal hydrolases L1 (UCHL-1) (a cytoplasmic ubiquitin C-terminal hydrolase) occurs on the mitochondria, resulting in harmful mitochondrial autophagy clearance. However, mitochondrial autophagy inhibition has a protective effect on NH2htau-induced synaptic degeneration and neuronal apoptosis [145]. Further studies have shown that Parkin overexpression was generally considered to have a neuroprotective effect in some tau diseases, mainly by promoting the degradation of misfolded aggregates of p-tau and proteasome/ubiquitin-dependent β-amyloid protein [146–148]. In an early neurodegenerative model of high intraocular pressure in rats, researchers found that overexpression of Parkin can prevent RGCs' loss and may attenuate mitochondrial autophagy [142]. Recent research has found that p-tau inserts into the mitochondrial membrane and impairs Parkin-mediated mitochondrial autophagy [149]. In addition, enhanced mitochondrial autophagy eliminated AD-related p-tau in human neuronal cells, indicating a possible interplay between p-tau and mitochondrial autophagy [150]. In conclusion, tau hyperphosphorylation is highly likely to result in RGCs' damage through the PINK1/Parkin pathway, leading to impaired mitochondrial autophagy and thereby contributing to the occurrence and progression of DRN.

#### 7.1.2. Mitochondrial Axonal Transport

Mitochondrial transport within the axon of neurons is crucial for maintaining the axon and its dysfunction can lead to neurodegenerative diseases [151]. Within neurons, mitochondrial axonal transport is typically divided into three types: anterograde transport, retrograde transport, and long-term stationary. The movement and stasis of mitochondria are in dynamic equilibrium and can respond rapidly to changes in axonal and synaptic physiology. Within the axon, mitochondrial movement is facilitated by two opposing proteins, motor proteins and kinesin, which move along microtubules [152]. Studies have shown that changes in KIF1A (kinesin that transports synaptic vesicle precursors), KIF5B (kinesin involved in mitochondrial transport and in the transport of synaptic vesicle precursors and membrane organelles), and motor protein (motor protein for retrograde axonal transport) can impair mitochondrial anterograde and retrograde axonal transport, leading to dysfunction of RGCs and DR [153].

Hyperphosphorylation of tau protein and impaired anterograde axonal transport of mitochondria were observed in K3 mice expressing the K369I tau mutation, leading to reduced mitochondrial quantity at synapses and neuronal dysfunction [135]. In addition, tau P301L selectively inhibited anterograde axonal transport of mitochondria without affecting retrograde transport and inhibited mitochondrial autophagy by suppressing Parkin translocation to mitochondria. This study also suggested that decreased transport mediated by motor proteins combined with persistent retrograde transport may decrease axonal mitochondria in tauopathy neurons, resulting in synaptic deficits [154]. The interaction between p-tau and JNK-interacting protein 1 (c-Jun N-JIP1) may affect anterograde axonal transport by directly interfering with the formation of motor protein–cargo complexes, rather than disrupting the binding between myosin heavy chain and microtubules [155]. Studies have also shown that p-tau protein at the AT8 site in the brain of AD patients reduced mitochondrial transport in axons, leading to axonal degeneration [156]. Moreover, some researchers have found that p-tau inhibits microtubule-dependent axonal/dendritic transport of mitochondria, leading to synaptic mitochondrial deprivation and synaptic starvation in the diabetic retina, resulting in synaptic loss and functional impairments of RGCs [34]. In summary, these findings suggest that p-tau interacts with c-Jun N-JIP1, disrupting the formation of protein–motor protein complexes and affecting mitochondrial axonal transport, leading to reduced mitochondrial content at synapses, which may contribute to synaptic loss and functional impairments of RGCs, and potentially influence the pathogenesis of DRN.

#### 7.1.3. Mitochondrial Dynamics

Mitochondrial dynamics are crucial for regulating the morphology, quantity, subcellular distribution characteristics, and function. Mitochondria change their shape and rearrange spatially through fission and fusion to adapt to the needs of the cell and maintain energy balance. Excessive fission or fusion can cause shortening or elongation, which in turn leads to mitochondrial dysfunction [157]. The structural changes of mitochondria are mainly regulated, maintained and remodeled by GTPase genes–dynamin-related protein 1 (Drp1), Fission 1 (Fis1), Mitofusins 1 and 2 (Mfn1 and Mfn2), and Optic atrophy 1 (Opa1) [158–160].

Increasing evidence suggested that abnormal mitochondrial dynamics, such as increased fission and decreased fusion, were early key factors found in neurodegenerative diseases [161]. Abnormally phosphorylated tau protein cleaved by the caspase family can synergize with Aβ to impair mitochondrial dynamics and function. Tau cleaved by Asp421 can induce mitochondrial fission alone, reduce mitochondrial motility, shorten mitochondrial length, and ultimately affect neuronal vitality [162]. Tau blocks Drp1 from locating to mitochondria by affecting the actin cytoskeleton, thereby changing the mitochondrial length and disrupting mitochondrial dynamics [163]. Drp1 interacted with Aβ and p-tau, which may lead to excessive mitochondrial fragmentation and mitochondrial and synaptic defects, ultimately resulting in neuronal damage [161]. In the cortex and hippocampus of double transgenic P301L tau and Drp1 knockout mice, reducing Drp1 and increasing mitochondrial fusion can prevent various tau-induced mitochondrial functional impairments [164]. In the early stages of tau pathology in THY–Tau22 mice, mitochondrial elongation has been observed, and Ca1 neurons in the hippocampus are rich in TauOs [165]. Another study showed that overexpression of hTau disrupted mitochondrial dynamics and led to neuronal dysfunction by increasing fusion proteins OPA1 and Mfn1/2, resulting in mitochondrial elongation [79]. It is worth noting that overexpression of the tau gene can increase the levels of fusion proteins Opa1, Mfn1, and Mfn2, leading to excessive accumulation and fusion of mitochondria, which can cause mitochondrial dysfunction. In the Opa1 knockout mouse model, mitochondrial fission increased in RGCs [166, 167]. Overexpression of Parkin increases the number of mitochondria in the axons of RGCs and reduces glutamate-induced mitochondrial fragmentation, thereby protecting RGCs [142]. In conclusion, the p-tau may influence mitochondrial dynamics by increasing the levels of fusion proteins Opa1, Mfn1, and Mfn2, ultimately leading to RGCs' damage.

#### 7.1.4. Mitochondrial Bioenergetics

Mitochondria provide energy to cells in the form of adenosine triphosphate (ATP) through oxidative phosphorylation (OXPHOS) [136]. OXPHOS is a coupled reaction where the energy released from the oxidation of substances in the body is supplied to adenosine diphosphate (ADP) and inorganic phosphate to synthesize ATP through the respiratory chain. Although the bioenergetics of mitochondria are crucial for cell survival, excessive generation of reactive oxygen species (ROS) induced by mitochondria can cause severe damage to cells. Excess ROS induces oxidative stress, leading to mitochondrial dysfunction, which can cause cell death and is implicated in the pathogenesis of many neurodegenerative diseases, including AD [168, 169]. Similarly, Tau (Ser-396, Ser-404, Thr-205, and Thr-231) overphosphorylation levels are observed to increase in mice lacking the detoxifying enzyme SOD2, indicating that mitochondrial oxidative stress can lead to p-tau [170]. Recently, related research has found that the P301L mutant tau can induce mitochondrial dysfunction and disrupt mitochondrial bioenergetics by inhibiting complex I activity, reducing mitochondrial membrane potential (ΔΨm) and ATP levels, and increasing ROS levels in pR5 mice (P301L tau mutant mice) [171–174]. Individuals with diabetes have decreased levels of retinal antioxidants such as manganese SOD, MnSOD, [130] and an overactive oxidative system such as increased NADPH oxidase 2, Nox2 [175], resulting in the production of large amounts of ROS, which can cause mitochondrial damage. Mitochondrial damage can cause the outer membrane to rupture by releasing cytochrome C, and cytochrome C can also activate apoptosis-related signaling pathways to induce early apoptosis of retinal neurons in patients with diabetes [129, 130, 176–178]. Therefore, p-tau may disrupt mitochondrial bioenergetics by inhibiting complex I activity, reducing ΔΨm, lowering ATP levels, and increasing ROS levels. Mitochondrial impairment can lead to the release of cytochrome C, causing mitochondrial outer membrane permeabilization, and subsequently activating apoptosis-related signaling pathways, thereby inducing early-stage retinal neuronal apoptosis in diabetes.

## 8. The p-Tau Interacts With Amyloid-Beta (Aβ) to Participate in DRN

The pathological changess in DRN include apoptosis of retinal neurons, including RGCs and amacrine cells. It has been found that the deposition of Aβ in the retina was related to the loss of RGCs [179, 180]. Moreover, targeted therapy through the Aβ pathway has shown the potential to reduce RGCs apoptosis and protect RGCs from degeneration. Specifically, the use of 18-7N (p1−4)-18gemini surfactants can inhibit the aggregation of Aβ40 peptide, which plays a role in the pathophysiological processes of glaucoma, leading to neurodegeneration and apoptosis of RGCs [181]. Therefore, it can be inferred that the deposition of Aβ may induce DRN. In addition, some researchers have also found that p-tau can cause DRN by disrupting synaptic and mitochondrial function in RGCs [34]. It is suggested that the p-tau may interact with Aβ and participate in the occurrence of DRN.

Research has found that Aβ within neurons can induce p-tau and cause Aβ toxicity [97, 182]. During the phosphorylation process, the Ser-262/Ser-356 sites on tau are first prephosphorylated by protein kinase C (PKC), CDK-5, and calcium–calmodulin–dependent protein kinase II (CaMKII), increasing the affinity of GSK-3β and other sites. Aβ can also accelerate the p-tau by mediating the activation of CDK-5 and GSK-3β, making other sites more easily recognized by GSK-3β and further promoting the phosphorylation process [183]. Aβ also participates in the formation of TauOs, which are a form of aggregation in the process of neurofibrillary tangle formation and can induce the binding of inflammatory factors with glial cells (astrocytes and microglia) and cause the death of glial cells [184]. Phosphorylated tau dissociated from microtubules, and Aβ activates CDK-5 and GSK3β, which act on phosphorylation sites to make them combine with other monomeric tau to form soluble oligomers. Aβ not only cleaves tau at Asp421 in the C-terminus by activating caspase-3 (CASP3) but also induces the production of a 17 kDa tau fragment by activating calpain-1 in hippocampal neurons. These tau fragments are more prone to aggregate and form soluble oligomers with misfolded tau [185, 186]. In addition, tau also affects the toxicity of Aβ. Tau transports Fyn (tyrosine–protein kinase) to dendritic spines, and Fyn phosphorylates NMDA, enhancing the binding of NMDA to postsynaptic density protein PSD95. NMDA and PSD95 form stable interactions, activating the signaling of the excitatory neurotransmitter Glu, which triggers the toxic effects of Aβ on neurons. Moreover, Fyn can also promote p-tau by activating tau phosphatase [187, 188].

On the other hand, the combined effects of Aβ and tau can also damage the neuroglial cells [189]. Both Aβ and tau can activate the microglia through the NF-κB and ERK signaling pathways, leading to the release of various inflammatory factors. Abnormal PTM of tau can also activate microglia via the PQBP1–cGAS–STING pathway, resulting in the release of inflammatory factors [190, 191]. In addition, the lack of the microglial division factor receptor CX3R1 may cause the microglia to take up and dissolve Aβ and TauOs, and the uptake of TauOs can further activate microglia to release inflammatory factors and promote the vicious cycle of tau hyperphosphorylation [191, 192]. Furthermore, microglia can also take up TauOs and secrete tau via exosomes, further promoting the pathological changes induced by tau protein. In summary, the effects of tau and Aβ on microglia constitute a vicious cycle. Studies have also shown that the TREM2 gene can guide microglia to take up and degrade the plaques induced by Aβ, thus alleviating the pathological propagation of tau [193]. Activation and aggregation of astrocytes are also related to the p-tau and the deposition of Aβ. Excessive deposition of Aβ and p-tau can promote the expression of transcription factor EB, thereby stimulating astrocytes to take up and degrade Aβ and extracellular abnormal PTM tau. At the same time, excessive deposition of Aβ and p-tau can also damage astrocytes by mediating the release of neuroinflammatory factors, thereby inhibiting the expression of transcription factor EB [194, 195].

The activation of the mammalian target of rapamycin (mTOR) leads to autophagy dysfunction. It modulates the metabolism of amyloid precursor protein (APP) and upregulates β- and γ-secretases, which enhances the production and deposition of Aβ. At the same time, mTOR activation accelerates the extent of tau hyperphosphorylation [196]. The generation of Aβ is regulated by an enzymatic metabolic process, where the membrane-bound endopeptidases β- and γ-secretases progressively cleave APP [197, 198]. Among these, β-secretase is the rate-limiting enzyme, while γ-secretase is the key enzyme, composed of APH1, PEN2, nicastrin, and presenilin (PS1 or PS2) [199, 200]. mTOR is an autophagy inhibitor that reduces the clearance rate of Aβ through the autophagy/lysosome system. mTOR may also interact with various signaling pathways, including PI3K/Akt, AMPK, GSK-3, and insulin/IGF-1, to regulate the production or clearance of Aβ [201–205]. Inhibition of mTOR activity induces autophagy, reduces Aβ aggregation, and enhances the Aβ clearance process [206].

mTOR signaling enhancement promotes the formation of tau pathology, while a reduction in mTOR signaling helps improve tau pathology [207]. A study found that diabetes activates mTOR by impairing insulin signaling, which increases tau hyperphosphorylation and promotes the onset of AD [208]. Overactivation of mTOR regulates autophagy, increasing levels of Aβ and p-tau. However, pretreatment with rapamycin (an mTOR inhibitor) can suppress mTOR overactivation, restore autophagic function, and effectively reduce hippocampal tau hyperphosphorylation, Aβ deposition, and cell apoptosis [209]. Rapamycin can also reduce tau phosphorylation at the Ser-214 site by modulating PKA. Phosphorylation at Ser-214 may trigger further phosphorylation of tau by other kinases, leading to tau hyperphosphorylation [210]. Rapamycin inhibits mTOR activity in a nondependent manner and enhances p-tau (Ser-396) phosphorylation through the PI3K pathway [211]. mTOR interacts with the PI3K signaling pathway to regulate PP2A and GSK-3-dependent phosphorylation of tau, with GSK-3β being antagonized by PP2A, thereby modulating multiple phosphorylation sites on tau [212]. In summary, rapamycin inhibits pathological changes associated with amyloid plaques and NFTs by promoting autophagy, and reducing mTOR signaling may emerge as a new strategy for treating tauopathies [207].

In addition, the interaction between Aβ and p-tau can directly lead to mitochondrial damage, which in turn accelerates the pathological progression of neurodegenerative diseases [161]. Overall, the mutual interaction between Aβ and tau exacerbates their respective toxic effects, providing new research directions for the treatment of neurodegenerative diseases.

## 9. Tau Protein Hyperphosphorylation and Inflammation Involved in DRN

Microglia are resident cells in the CNS that can be activated and release inflammatory factors due to high blood sugar, abnormal blood lipids, and other reasons, leading to harmful neuroinflammation and subsequently affecting Aβ and tau accumulation, causing neuronal damage and ultimately aggravating neurodegeneration [213–215]. Research has found that [216] p-tau promotes neuronal death by activating both the RIPK1/RIPK3/MLKL-mediated necroptosis pathway and the nuclear factor kappa B (NF-κB) inflammatory signaling cascade, and p-tau can promote neuronal death through NF-κB signaling pathway, which stimulates the cellular autonomous overexpression of cytokines and chemokines in neuronal cells, both of which require the involvement of RIPK1–RIPK3–MLKL axis. It also indicated that necrotic apoptosis was accompanied by an NF-κB signaling pathway that mediates upregulation of proinflammatory factor transcription, while microglia were activated by proinflammatory factors, leading to neuronal cell death. Related studies have also confirmed that the NF-κB signaling pathway was a necessary pathway for p-tau–induced cytokine transcription in neuronal cells [216, 217]. It has also been shown that purified recombinant truncated tau induced morphological transformation in microglia and may release proinflammatory cytokines by activating NF-κB signaling pathway (IL-1β, IL-6, and TNF-α) [218]. p-tau may induce the expression of inflammatory cytokines such as IL-1β, IL-6, and TNF-α through the NF-κB signaling pathway [219]. In addition, in a mouse model of tau-mediated neurodegeneration, inactivation of microglia can weaken neuroinflammation and tau-mediated neurodegeneration [220, 221]. Multiple studies have found that the inflammatory response caused by microglia played a major role in the DRN [15, 222, 223]. Microglia can be divided into M1 microglia and M2 microglia. M1 microglia can induce neurotoxicity by expressing high levels of IL-1β, IL-23, TNFα, and IL-6, while M2 microglia, also known as phagocytic microglia, can clear necrotic apoptotic cells by expressing high levels of IL-10. The regulation of M1 and M2 phenotypic microglia *in vivo* depends on the progression of the disease. M1 microglia, when induced by interferon-gamma (INF-γ) and lipopolysaccharide (LPS), release proinflammatory cytokines (IL-1β, IL-6, and TNF-α), which damage neurons by accelerating the inflammatory response [224]. Therefore, an excessive presence of abnormally regulated M1 microglia can lead to various neurodegenerative diseases such as DRN, AD, Parkinson's disease, and others [225–227]. In the normal retina, microglia play the role of tissue macrophages and participate in regulating the growth, development, and function of retinal neurons and blood vessels [228]. In the pathogenesis of DR, oxidative stress caused by hyperglycemia triggers the expression of toll-like receptor-2 (TLR-2), TLR-4, and NF-κB through ROS. Meanwhile, due to the expression of NF-κB, activated microglia accumulate around ischemic microvessels, causing chronic inflammatory response [228, 229]. In addition, activated microglial cells release cytotoxic factors, leading to increased production of inflammatory cytokines and ROS, which participate in neuronal damage in neurodegenerative diseases [230–232].

## 10. Targeted Therapy for Tau Protein Hyperphosphorylation

Tau protein hyperphosphorylation is considered a key pathological feature of various neurodegenerative diseases. In recent years, targeted therapies for tau protein hyperphosphorylation have been continuously developed, offering new possibilities for disease intervention. The following are some of the most promising therapeutic strategies.

Recent studies have identified *cis-*p-tau as an early pathogenic tau conformation capable of initiating and propagating neurodegenerative pathology [233]. A study found that after TBI in mice and stress *in vitro*, neurons significantly produce *cis*-p-tau, which triggers a pathogenic process termed “cistauosis” both *in vitro* and *in vivo*. This process leads to tau-mediated neurodegeneration and brain atrophy. The use of *cis*-p-tau antibodies effectively neutralizes *cis*-p-tau, halts cistauosis, prevents secondary brain injury after TBI, and inhibits the progression of neurodegeneration and brain atrophy [87]. The unique prolyl isomerase Pin1 catalyzes the *cis*–*trans* isomerization of certain pSer/Thr-Pro motifs in a phosphorylation-dependent manner [234–236]. It plays a crucial role in converting the phosphorylated Thr-231-Pro motif in p-tau from the pathogenic *cis* conformation to the physiological *trans* conformation, thereby restoring the function of p-tau and protecting the brain from tau-mediated neurodegeneration in AD [237]. *Cis*-p-tau antibody antibodies could be further developed as potential therapeutic targets for neurodegenerative diseases, but further research and evaluation are needed.

mTOR is a key signaling pathway molecule involved in regulating cellular autophagy and protein metabolism. Overactivation of mTOR may be an important cause of tau hyperphosphorylation [238]. Related studies have also shown that rapamycin, an mTOR inhibitor, can suppress mTOR activity and reduce the levels of total tau protein both *in vitro* and *in vivo* [239–241]. Rapamycin enhances cellular autophagy, which promotes the clearance of p-tau, thereby reducing tau-induced neurotoxicity [242–244]. It has also been suggested that rapamycin may inhibit tau synthesis, further reducing tau accumulation [245]. However, rapamycin-induced autophagy primarily clears hyperphosphorylated and insoluble tau, as soluble tau, being dispersed throughout the cell, is less likely to be degraded by the autophagic pathway [242]. In addition, rapamycin can regulate the cAMP-dependent kinase (PKA) pathway to reduce tau phosphorylation at the Ser-214 site, providing a potential new mechanism for reducing or preventing tau hyperphosphorylation [210]. Therefore, rapamycin regulates tau accumulation and toxicity through multiple pathways and may serve as a potential strategy for treating tau pathology.

MAPK and ERK signaling pathways are widely recognized as key contributors to the abnormal phosphorylation of tau protein. Activation of these pathways can lead to hyperphosphorylation of tau at multiple sites, promoting its aggregation and inducing neurotoxicity [246, 247]. Activation of p38-MAPK was involved in neuronal responses to various stresses and is closely associated with hyperphosphorylation of tau protein in AD [248, 249]. One study found that long-term treatment with an ERK2 small molecule inhibitor significantly reduced the levels of abnormally p-tau species in JNPL3 transgenic mice expressing the P301L mutant tau, preventing severe motor deficits [250]. Furthermore, ERK2 inhibition was shown to reduce levels of abnormally phosphorylated tau in mouse models of tauopathies [90].

In recent years, several common therapeutic approaches to inhibit tau aggregation have emerged. Phosphorylated tau-targeting small molecules, such as proteolysis targeting chimeras (PROTACs), represent a novel strategy for proteasomal degradation via the ubiquitin–proteasome system. This approach utilizes chemical knockdown to degrade toxic tau species through the ubiquitin–proteasome pathway [251]. There is also a nanocomposite material (CeNC/IONC/MSN-T807) loaded with methylene blue (MB) that has a high binding affinity for p-tau. Although several small molecule therapies targeting tau-related diseases have been developed, their safety and efficacy as potential new drugs still require further investigation [252].

## 11. Discussion

**Figure 2:**
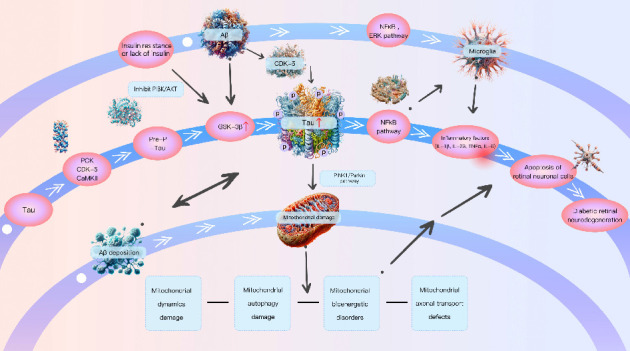
Interaction of tau and DRN. Tau is first prephosphorylated by PKC, CDK-5 and CaMKII, in addition, insulin resistance and lack of insulin inhibit the PI3K/AKT pathway, activating GSK-3β and causing hyperphosphorylation of tau protein. Aβ accelerates the p-tau by mediating the activation of CDK-5 and GSK-3β; Aβ deposition and p-tau interact with each other and can directly induce mitochondrial damage, while p-tau can also induce mitochondrial damage through the PINK1/Parkin signaling pathway, ultimately leading to apoptosis of retinal neuronal cells and DRN. Both Aβ and tau can activate microglia through NF-κB and ERK signaling pathways, releasing various inflammatory factors (IL-1β, IL-23, TNF-α, and IL-6), eventually leading to apoptosis of retinal neuronal cells and DRN.

This article review indicates that p-tau plays a crucial role in the pathogenesis of DRN. However, its underlying molecular mechanisms are not yet clear. p-tau may cause mitochondrial damage, such as insulin resistance or lack of insulin, mitophagy impairment, axonal transport disruption, mitochondrial bioenergetics, mitochondrial dynamics, Abeta toxicity, and inflammation involved in DRN (for more information on the mechanism diagram, please refer to Figure 2). Despite many breakthroughs in the DR field, further research is still needed for precise diagnostic methods and more effective treatments for DRN. Pathogenic tau molecules are excellent therapeutic targets for neurodegenerative diseases, using various strategies such as lowering levels, altering post translational modifications, or blocking tau hyperphosphorylation. In current commonly used therapeutic methods to inhibit tau aggregation, the use of small molecules is an important approach. Overall, understanding the pathological and physiological mechanisms of p-tau in the pathogenesis of DRN will aid in the future treatment of DRN, and tau may also become an increasingly important therapeutic target in the future.

## Figures and Tables

**Table 1:** Distribution of tau exons in the nervous system.

Exon	Retina	PNS	CNS
1	No	Yes	Yes
2	No	No	Yes
3	No	No	Yes
4	No	No	Yes
4A	Yes	Yes	No
5	No	Yes	Yes
7	No	Yes	Yes
9	No	Yes	Yes
10	No	No	Yes
11	No	Yes	Yes
12	No	Yes	Yes
13	No	Yes	Yes

## Data Availability

The authors have nothing to report.

## Nomenclature

DRN: Diabetic retinal neurodegeneration
DR: Diabetic retinopathy
PHF: Paired helical filaments
RGCs: Retinal ganglion cells
p-tau: Hyperphosphorylated Tau
OCT: Optical coherence tomography
RNFL: Retinal nerve fiber layer
GCL: Ganglion cell layer
IPL: Inner plexiform layer
CNS: Central nervous system
PNS: Peripheral nervous system
MBD: Microtubule-binding domain
AD: Alzheimer's disease
PTM: Posttranslational modification.
